# Degradation of Polymers and Heavy Metals in Waste Drilling Fluid by Sulfur-Doped BiOBr_0.5_Cl_0.5_ Photocatalysts

**DOI:** 10.3390/gels11090684

**Published:** 2025-08-27

**Authors:** Tengfei Dong, Guancheng Jiang, Sihe Jiang, Yinbo He, Lili Yang

**Affiliations:** 1 College of Science, China University of Petroleum (Beijing), 18 Fuxue Road, Changping, Beijing 102249, China; 2 College of Petroleum Engineering, China University of Petroleum (Beijing), 18 Fuxue Road, Changping, Beijing 102249, China18210845055@163.com (S.J.);

**Keywords:** sulfur doping, heavy metals, waste drilling fluids, active species, oxygen vacancies

## Abstract

Waste drilling fluids represent a complex gel–colloidal system containing structurally stable polymeric networks and heavy-metal ions that can cause tremendous damage to the ecosystem. The current disposal methods, like solidification/landfills, formation reinjection, and chemical treatment, commonly suffer from high secondary pollution risks, poor resource recovery, and incomplete detoxification. This paper developed a photocatalytic approach to complex gel system treatment by hydrothermally synthesizing a novel sulfur-doped, oxygen-vacancy-modified 3D flower-like xS-BiOBr_0.5_Cl_0.5_ structure which effectively narrowed the bandgap of BiOX and thus significantly enhanced its catalytic activity. The chemical composition, morphology, specific surface areas, and bandgaps of the materials were characterized. The photocatalytic performance and cyclic stability of the materials were measured, and 0.5S-BiOBr_0.5_Cl_0.5_ showed the best photocatalytic performance. The rhodamine B(RhB) degradation and polymer degradation efficiencies of 0.5S-BiOBr_0.5_Cl_0.5_ were up to 91% and 79%, respectively, while the Hg(II), Cr(VI), and Cr(III) reduction efficiencies of the material were up to 48.10%, 96.58%, and 96.41%, respectively. The photocatalytic mechanism of the xS-BiOBr_0.5_Cl_0.5_ materials was evaluated through an oxygen vacancy analysis, active species capture experiments, and density functional theory (DFT) computations. Overall, the xS-BiOBr_0.5_Cl_0.5_ materials can provide a low-cost and harmless treatment method for waste drilling fluids and promote the “green” development of oil and gas.

## 1. Introduction

Drilling fluids play important roles in the drilling process, including carrying drill cuttings, cooling the drill bit, and balancing the formation pressure. Waste drilling fluids form structurally stable polymeric gels containing large quantities of hazardous polymers and heavy metals that can cause serious harm to the environment and human health. The traditional treatment methods for waste drilling fluids are complicated and costly, and they can cause secondary pollution. Therefore, environmentally friendly, inexpensive, and highly effective treatment methods for drilling fluids are urgently needed [1,2,3,4].

Photocatalysis is considered to be one of the most promising technologies for solving the current environmental pollution problems because it is a highly effective technology with no toxicity or secondary pollution. The efficiency of a photocatalytic process depends on the efficiency of the photocatalyst [5,6,7,8]. **TiO_2_ has been widely used as a photocatalyst.** However, due to its large bandgap, it can only be excited by ultraviolet light (which only accounts for 5% of the solar spectrum), and the lifetime of its photogenerated electrons and holes is short. The development of visible-light-driven photocatalysts with the efficient utilization of visible light and extended lifetimes for the photogenerated electrons and holes is a current trend in photocatalysis research [9,10,11,12,13,14]. The valence band of a bismuth-based photocatalyst is formed through the hybridization of Bi 6s and O 2p orbitals. Therefore, the valence band of the photocatalyst is elevated, and the width of the bandgap of the material is reduced, making the material excitable using visible light [15,16,17,18,19,20].

BiOX (X = Cl, Br, or I) is a prominent class of bismuth-based photocatalyst characterized by a chlorophorbide (PdFCl)-type crystalline structure. It crystallizes into a tetragonal system with the P4/nmm space group. The defining structural feature is its layered arrangement along the c-axis, consisting of positively charged [Bi_2_O_2_]^2+^ slabs alternately stacked with double layers of halide anions (X^−^ sheets). This unique anisotropic structure creates pronounced charge separation between the cationic [Bi_2_O_2_]^2+^ layers and the anionic X^−^ sheets. This inhomogeneous charge distribution induces a strong dipole moment and significant lattice polarization, primarily affecting the Bi 6s and O 2p orbitals. Crucially, the interlayer space provides ample room for this polarization to develop fully. Consequently, a robust internal static electric field (IEF) is generated perpendicular to the layers (along the c-axis). This built-in field is pivotal for photocatalysis, as it drastically enhances the spatial separation of the photogenerated electrons and holes by driving electrons towards the X^−^ layers and holes towards the [Bi_2_O_2_]^2+^ slabs, effectively suppressing recombination and significantly boosting the photocatalytic efficiency [21,22,23,24]. The magnitude of this IEF and the resultant activity can be tuned using the halogen ion (Cl, Br, I).

There are various strategies for narrowing the bandgap of a photocatalyst, among which defect engineering is the most important strategy for achieving a high photocatalytic efficiency [25,26,27,28,29]. Oxygen vacancies can act as charge-trapping centers and adsorption sites for photoexcitation, and their introduction into a photocatalyst is the most convenient method to prevent the complexation of photogenerated electrons and holes [30,31,32,33,34].

Sulfur doping represents a particularly effective strategy for enhancing the visible light response of BiOX photocatalysts by narrowing their bandgaps. Unlike early reports on nitrogen-doped TiO_2_ (where nitrogen substituted lattice oxygen to enable visible light excitation [35,36,37,38,39,40]), sulfur dopants primarily modify the electronic structure through S 3p orbital hybridization with the valence band (VB). This hybridization elevates the VB maximum and introduces localized states near the VB edge, effectively reducing the bandgap energy. Consequently, sulfur-doped BiOX materials exhibit significantly enhanced visible light absorption and photocatalytic activity.

In this study, sulfur-doped and oxygen-vacancy-modified three-dimensional flower-like xS-BiOBr_0.5_Cl_0.5_ materials were synthesized using a hydrothermal method. Under visible light excitation, the materials were able to degrade polymers and heavy metals that are commonly found in waste drilling fluids, providing a low-cost, simple, and harmless treatment method for waste drilling fluids and promoting the “green” development of the oil and gas industry.

## 2. Results and Discussion

### 2.1. The Crystal Structure of xS-BiOBr_0.5_Cl_0.5_ Materials

**Figure 1:**
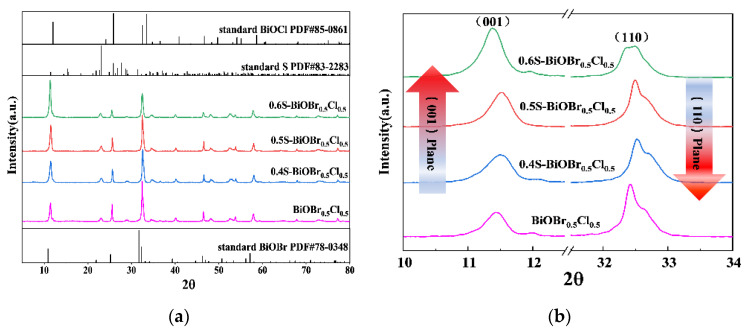
XRD spectra of xS-BiOBr_0.5_Cl_0.5_ materials (**a**,**b**).

The crystal structure of the xS-BiOBr_0.5_Cl_0.5_ materials was analyzed using X-ray diffraction spectroscopy (XRD), and the results are shown in Figure 1. Due to the partial substitution of Br^−^ with Cl^−^, the diffraction peaks of BiOBr_0.5_Cl_0.5_ were shifted to higher angles relative to those of BiOBr, which indicated that the substitution caused the lattice parameter of BiOBr_0.5_Cl_0.5_ to be lower than that of BiOBr. The diffraction peaks of the xS-BiOBr_0.5_Cl_0.5_ materials were consistent with those of BiOBr, suggesting that the crystal structure of the materials was consistent with that of BiOBr, although the materials contained sulfur dopants. All of the diffraction peaks in the XRD spectra of the xS-BiOBr_0.5_Cl_0.5_ materials were sharp, and there were no impurity peaks in the XRD spectra, demonstrating the good crystallinity and purity of the materials. With an increasing sulfur concentration in the xS-BiOBr_0.5_Cl_0.5_ materials, the intensity of the diffraction peak corresponding to the (001) plane increased, while the intensity of the diffraction peak corresponding to the (110) plane gradually decreased, indicating that crystals in the materials gradually grew along the (110) plane with an increasing concentration of sulfur dopants in the materials [41].

### 2.2. The Morphology and Thickness of the xS-BiOBr_0.5_Cl_0.5_ Materials

**Figure 2:**
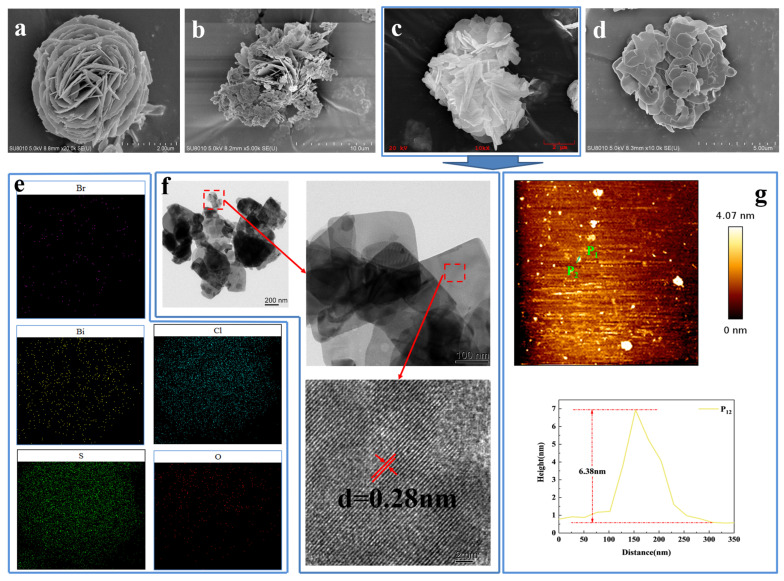
SEM images of BiOBr_0.5_Cl_0.5_ (**a**) and xS-BiOBr_0.5_Cl_0.5_ materials (**b**–**d**). EDS mapping images of 0.5S-BiOBr_0.5_Cl_0.5_ (**e**). TEM and HRTEM images of 0.5S-BiOBr_0.5_Cl_0.5_ (**f**). The AFM image and the corresponding height-distribution profile of 0.5S-BiOBr_0.5_Cl_0.5_ (**g**).

Scanning electron microscopy (SEM) was used to investigate the morphology of the xS-BiOBr_0.5_Cl_0.5_ materials. As shown in Figure 2a, BiOBr_0.5_Cl_0.5_ consists of three-dimensional flower-like microspheres assembled of two-dimensional interleaved nanosheets with a diameter of about 2 µm. With the addition of thiourea to BiOBr_0.5_Cl_0.5_, the three-dimensional flower-like structure gradually loosened, the number of interleaved-nanosheet layers decreased, and the spacing between the interleaved-nanosheet layers increased, all of which contributed to efficient electron transfer on the surface of the resulting xS-BiOBr_0.5_Cl_0.5_ material (Figure 2b–d). When the concentration of thiourea added was 0.6 mmol (Figure 2d), the flower-like structure completely disassembled, and the resulting lamellar structure was stacked along the *z*-axis [42,43]. The surface elemental composition of the 0.5S-BiOBr_0.5_Cl_0.5_ microspheres was analyzed using energy-dispersive X-ray spectroscopy (EDS) elemental mapping (Figure 2e), which showed that Bi, O, S, Cl, and Br were evenly distributed on the surface of the microspheres.

The lattice spacing of 0.5S-BiOBr_0.5_Cl_0.5_, which was examined through transmission electron microscopy (TEM) and high-resolution transmission electron microscopy (HRTEM), was 0.28 nm (Figure 2f), which matched the (110) lattice spacing of BiOBr [44]. Therefore, the introduction of sulfur into BiOBr_0.5_Cl_0.5_ did not cause a distinct difference between the crystal structure of BiOBr_0.5_Cl_0.5_ and that of BiOBr, which was consistent with the XRD results.

The thickness of the 0.5S-BiOBr_0.5_Cl_0.5_ nanosheets was measured through contact-mode atomic force microscope (AFM) direct imaging. Figure 2g shows that 0.5S-BiOBr_0.5_Cl_0.5_ is spherical and nanostructured. A random 0.5S-BiOBr_0.5_Cl_0.5_ nanosheet was chosen to measure the thickness of the material. On the basis of the height-distribution profile of the nanosheet, the nanosheet’s thickness was about 6.38 nm, which was eight times greater than the unit cell size of BiOBr (0.81 nm; PDF#73-2061).

### 2.3. Specific Surface Areas of the xS-BiOBr_0.5_Cl_0.5_ Materials

**Figure 3:**
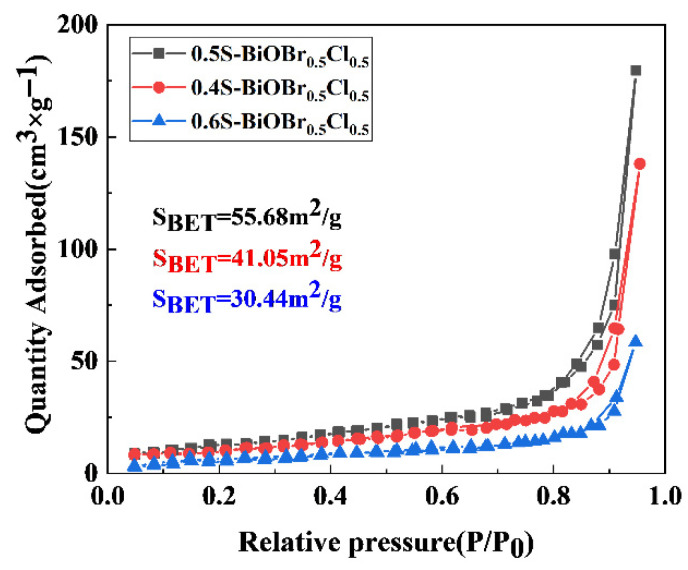
Nitrogen adsorption–desorption isotherms of xS-BiOBr_0.5_Cl_0.5_ materials.

The specific surface areas of the xS-BiOBr_0.5_Cl_0.5_ materials were measured through a Brunauer–Emmett–Teller (BET) analysis. As shown in Figure 3, the specific surface areas of 0.4S-BiOBr_0.5_Cl_0.5_, 0.5S-BiOBr_0.5_Cl_0.5_, and 0.6S-BiOBr_0.5_Cl_0.5_ are 41.05 m^2^/g, 55.68 m^2^/g, and 30.44 m^2^/g, respectively. With an increasing concentration of thiourea added to BiOBr_0.5_Cl_0.5_, the specific surface areas of the xS-BiOBr_0.5_Cl_0.5_ materials increased and then decreased, which was consistent with the SEM results (Figure 2). The addition of thiourea to BiOBr_0.5_Cl_0.5_ gradually loosened the flower-like structure of the material, and the three-dimensional flower-like structure of BiOBr_0.5_Cl_0.5_ was completely damaged when an excessive amount of thiourea was added to the material, leading to stacking of the nanosheets of the material along the *z*-axis, a reduction in the specific surface area of the material, an increase in the complexation of photogenerated electrons and holes, and a reduction in the photocatalytic efficiency of the material.

### 2.4. Surface Chemical Composition and Surface-Chemical-Element Valence States of the xS-BiOBr_0.5_Cl_0.5_ Materials

**Figure 4:**
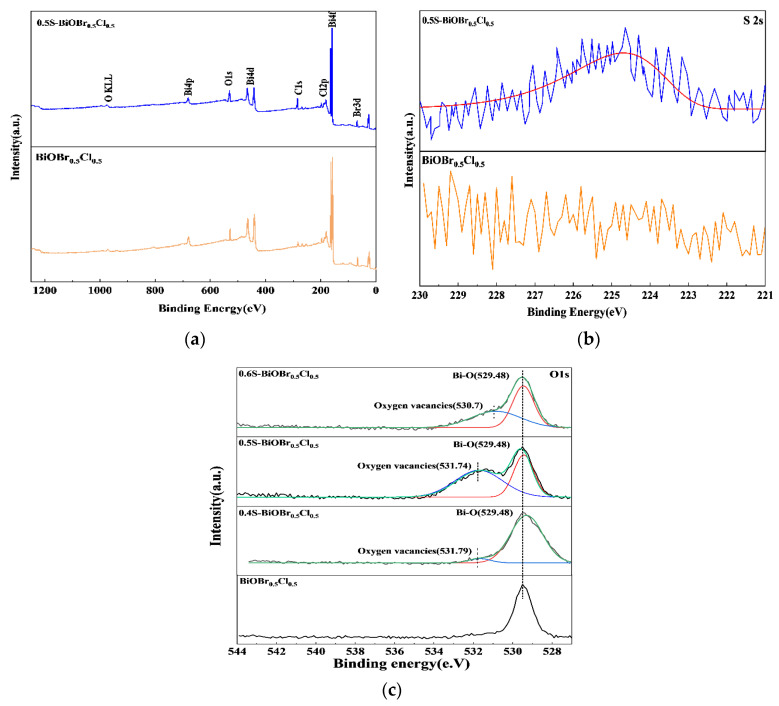
XPS survey spectra (**a**) and high-resolution S 2s spectra (**b**) of BiOBr_0.5_Cl_0.5_ and 0.5S-BiOBr_0.5_Cl_0.5_. High-resolution O 1s spectra of BiOBr_0.5_Cl_0.5_ and xS-BiOBr_0.5_Cl_0.5_ materials (**c**).

The surface elemental composition of BiOBr_0.5_Cl_0.5_ and 0.5S-BiOBr_0.5_Cl_0.5_ and the valence states of the chemical elements on the surfaces of the materials were investigated through X-ray photoelectron spectroscopy (XPS). The XPS survey spectra of BiOBr_0.5_Cl_0.5_ and 0.5S-BiOBr_0.5_Cl_0.5_ (Figure 4a) demonstrated that the surfaces of the materials consisted of the same elements (Bi, O, Br, and Cl). However, the peak corresponding to the sulfur element was not detected in the XPS survey spectrum of 0.5S-BiOBr_0.5_Cl_0.5_ since the S 2s and Bi 4f peaks in the spectrum were highly overlapping. The existence of sulfur dopants in 0.5S-BiOBr_0.5_Cl_0.5_ was confirmed by the high-resolution S 2s spectrum of the material (Figure 4b). A signal corresponding to S^2−^ was observed in the high-resolution S 2s spectrum of 0.5S-BiOBr_0.5_Cl_0.5_ at 225.3 eV, suggesting that sulfur dopants existed in the material in the form of S^2−^.

The high-resolution O 1s spectra of xS-BiOBr_0.5_Cl_0.5_ materials were deconvoluted into the characteristic peaks of lattice oxygen (529.48 eV) and adsorbed oxygen (530.70 eV).The characteristic peak of adsorbed oxygen was not found in the high-resolution O 1s spectrum of BiOBr_0.5_Cl_0.5_ due to the lack of oxygen vacancies on the surface of the material. The lack of oxygen vacancies prevented the effective adsorption of oxygen molecules onto the surface of the material. With the introduction of sulfur into BiOBr_0.5_Cl_0.5_, the characteristic peak of adsorbed oxygen appeared in the high-resolution O 1s spectra of xS-BiOBr_0.5_Cl_0.5_ materials. The largest area of the adsorbed oxygen peak was observed in the high-resolution O 1s spectrum of 0.5S-BiOBr_0.5_Cl_0.5_, proving that the number of oxygen vacancies on the surface of 0.5S-BiOBr_0.5_Cl_0.5_ was greater than that on the surfaces of 0.4S-BiOBr_0.5_Cl_0.5_ and 0.6S-BiOBr_0.5_Cl_0.5_ [45,46,47].

### 2.5. The Optical Properties of the xS-BiOBr_0.5_Cl_0.5_ Materials

**Figure 5:**
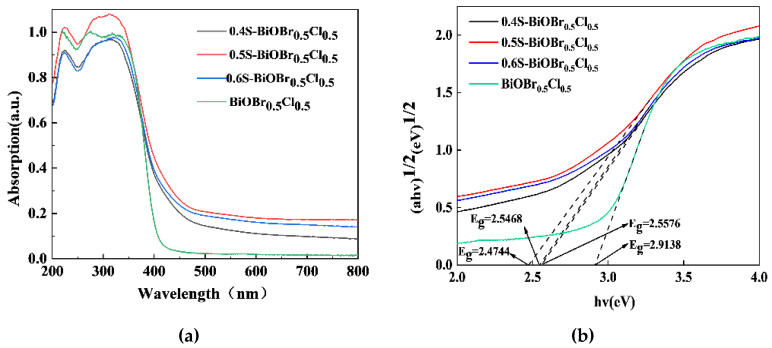
UV–vis absorption spectra (**a**) and bandgaps (**b**) of xS-BiOBr_0.5_Cl_0.5_ materials.

The optical properties of the xS-BiOBr_0.5_Cl_0.5_ materials were evaluated through UV–vis spectroscopy in the wavelength range of 200–800 nm. The UV–vis absorption spectra of the xS-BiOBr_0.5_Cl_0.5_ materials were redshifted relative to the UV–vis absorption spectrum of BiOBr_0.5_Cl_0.5_ (Figure 5a), and the materials were photoresponsive in the visible light wavelength range, with 0.5S-BiOBr_0.5_Cl_0.5_ being the most photoresponsive among the xS-BiOBr_0.5_Cl_0.5_ materials. The bandgaps of the materials were calculated by extrapolating the (αhν)^1/2^ vs. hν plots of the materials (xS-BiOBr_0.5_Cl_0.5_ materials were semiconductors with indirect bandgaps) [48]. As shown in Figure 5b, the bandgaps of BiOBr_0.5_Cl_0.5_, 0.4S-BiOBr_0.5_Cl_0.5_, 0.5S-BiOBr_0.5_Cl_0.5_, and 0.6S-BiOBr_0.5_Cl_0.5_ are 2.91 eV, 2.56 eV, 2.47 eV, and 2.54 eV, respectively. These results indicated that the homogeneous distribution of sulfur dopants in the crystal structure of BiOBr_0.5_Cl_0.5_ enabled the xS-BiOBr_0.5_Cl_0.5_ material to have a narrow bandgap due to an increase in the binding affinity between S^2−^ and Bi^3+^. However, due to the excessive concentration of sulfur dopants in 0.6S-BiOBr_0.5_Cl_0.5_, the bandgap of the material was narrower than those of 0.4S-BiOBr_0.5_Cl_0.5_ and 0.5S-BiOBr_0.5_Cl_0.5_, inducing the recombination of photogenerated electron–hole pairs in the material and negatively affecting the photocatalytic performance of the material.

### 2.6. The Photocatalytic Activity of the xS-BiOBr_0.5_Cl_0.5_ Materials

**Figure 6:**
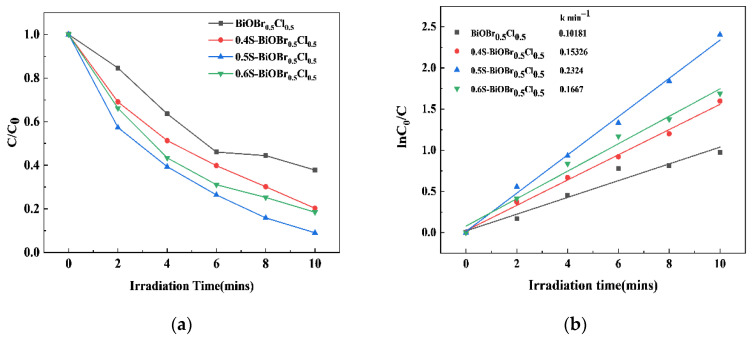
Efficiencies (**a**) and apparent rate constants (**b**) for the photocatalytic degradation of RhB by the xS-BiOBr_0.5_Cl_0.5_ materials.

The photocatalytic activity of the xS-BiOBr_0.5_Cl_0.5_ materials under visible light irradiation was evaluated using RhB as a contaminant. As depicted in Figure 6a, the RhB degradation efficiency of 0.5S-BiOBr_0.5_Cl_0.5_ is greater than that of 0.4S-BiOBr_0.5_Cl_0.5_ and 0.6S-BiOBr_0.5_Cl_0.5_, reaching 91.06% within 10 min. In comparison, the RhB degradation efficiency of BiOBr_0.5_Cl_0.5_ reached 62.23% within 10 min, suggesting that the introduction of oxygen vacancies and sulfur dopants into BiOBr_0.5_Cl_0.5_ improved the photocatalytic performance of the resulting xS-BiOBr_0.5_Cl_0.5_ materials. The reaction kinetics of the RhB degradation by the xS-BiOBr_0.5_Cl_0.5_ materials was described using a pseudo-first-order kinetic model:

ln(*C*_0_/*C*) = *kt* (1)

where *C*_0_ represents the initial concentration (mg/L), *C* is the concentration at the reaction time t (mg/L), k is the rate constant (min−1), and t is the reaction time (min).

Figure 6b shows that the degradation of RhB by the xS-BiOBr_0.5_Cl_0.5_ materials follows pseudo-first-order kinetics. The apparent rate constant for the degradation of RhB by 0.5S-BiOBr_0.5_Cl_0.5_ was 0.2324/min, which was 2.28 times higher than that for the degradation of RhB by BiOBr_0.5_Cl_0.5_, further demonstrating the superior photocatalytic performance of the photocatalyst with sulfur dopants and oxygen vacancies.

### 2.7. The Cyclic Stability of the xS-BiOBr_0.5_Cl_0.5_ Materials

**Figure 7:**
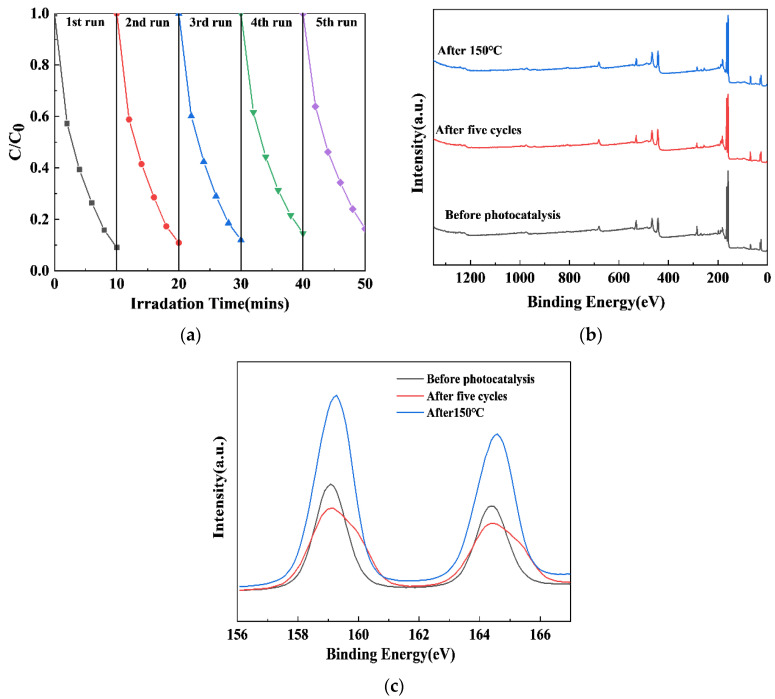
The photocatalytic performance of 0.5S-BiOBr_0.5_Cl_0.5_ over five RhB degradation cycles (**a**). XPS survey spectra (**b**) and high-resolution Bi 4f spectra (**c**) of 0.5S-BiOBr_0.5_Cl_0.5_ measured after five RhB degradation cycles or 150 °C heating for 1 h under a nitrogen atmosphere.

The results of the experiments on the cyclic stability of the xS-BiOBr_0.5_Cl_0.5_ materials are shown in Figure 7. The RhB degradation efficiency of 0.5S-BiOBr_0.5_Cl_0.5_ reduced from 91.69% in the first cycle to 83.72% by the fifth cycle (Figure 7a), which was due to the loss of the material during collection and purification. Therefore, 0.5S-BiOBr_0.5_Cl_0.5_ maintained excellent RhB degradation efficiency for five RhB degradation cycles. The stability of 0.5S-BiOBr_0.5_Cl_0.5_ after five RhB degradation cycles and after heating at 150 °C for 1 h under a nitrogen atmosphere was evaluated by measuring the XPS survey spectrum and the high-resolution Bi 4f spectrum of the material. After five RhB degradation cycles or 150 °C heating, no obvious changes were detected in the XPS survey spectrum of the material (Figure 7b), while only the heights of the peaks in the Bi 4f spectrum of the material were altered (Figure 7c), suggesting that there were no changes in the valence states of the surface chemical elements of the material. It can be concluded that 0.5S-BiOBr_0.5_Cl_0.5_ is a stable and recyclable photocatalyst with a favorable photocatalytic performance.

### 2.8. The Photocatalytic Reduction of Heavy-Metal Ions by xS-BiOBr_0.5_Cl_0.5_ Materials

**Figure 8:**
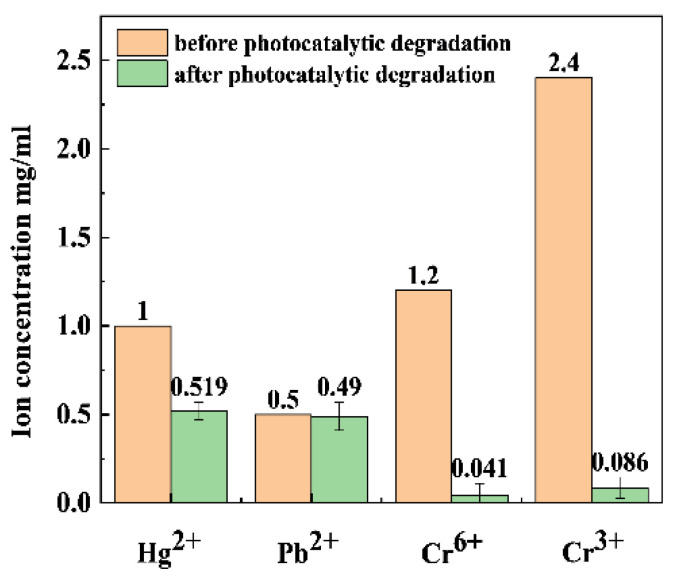
Photocatalytic reduction of heavy-metal ions by xS-BiOBr_0.5_Cl_0.5_ materials.

Various chemicals are added to drilling fluids to ensure the stability of the fluids. Unfortunately, many of these chemicals contain heavy-metal ions. At the same time, some of the metal ions in the formation will be dissolved into the drilling fluids, where Hg(II), Pb(II), Cr(VI), and Cr(III) ions are the main heavy-metal ions in the fluids [49]. In this study, 0.5S-BiOBr_0.5_Cl_0.5_ was used to reduce the above heavy-metal ions under visible light irradiation. As shown in Figure 8, the Hg(II), Pb(II), Cr(VI), and Cr(III) reduction efficiencies of 0.5S-BiOBr_0.5_Cl_0.5_ are 48.10%, 2.00%, 96.58%, and 96.41%, respectively, demonstrating the good Cr(VI) and Cr(III) reduction efficiencies of the material.

### 2.9. Photocatalytic Degradation of Polymers by the xS-BiOBr_0.5_Cl_0.5_ Materials

**Figure 9:**
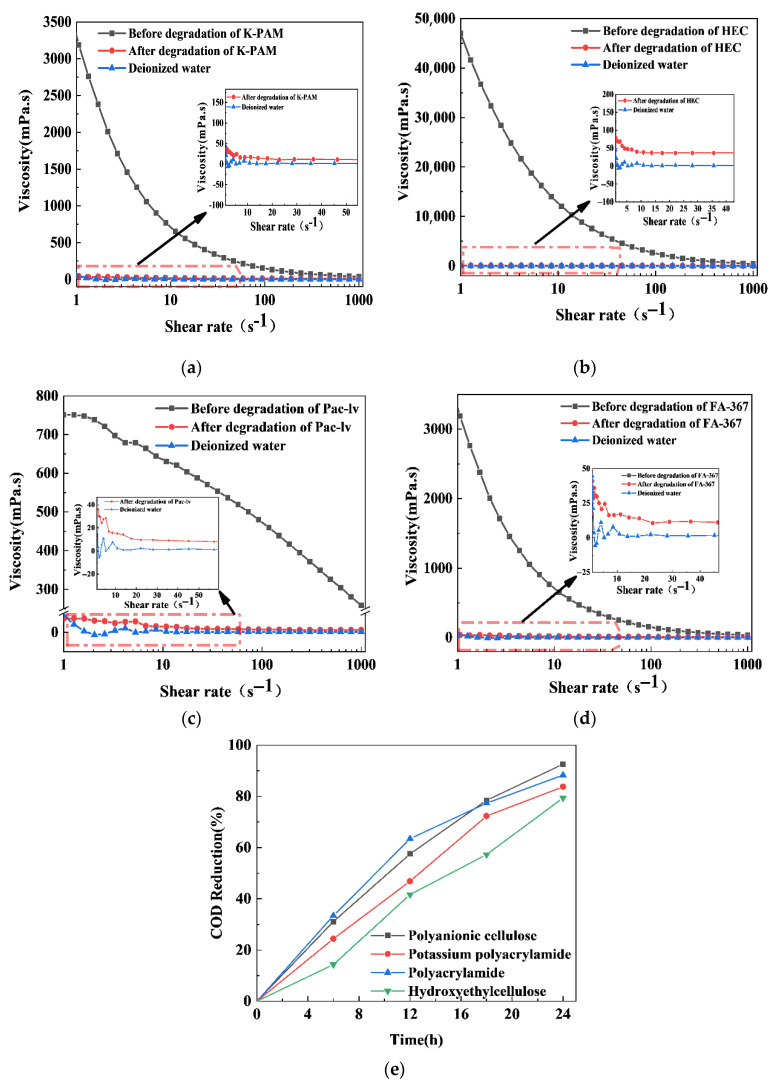
The viscosities of K-PAM (**a**), HEC (**b**), PAC-LV (**c**), and FA-367 (**d**) solutions measured at different shear rates before and after the photocatalytic degradation of the polymers. Rates of COD reduction in the polymer solutions (**e**).

Polyanionic cellulose, polyacrylamide, potassium polyacrylamide, and hydroxyethyl cellulose are key components in drilling fluids, functioning as loss reduction agents, wellbore stabilizers, rock-coating agents, and shear enhancement additives, respectively. These polymers are primarily responsible for building and maintaining the gel structure and rheological properties of the fluid [50]. As shown in Figure 9a–d, the viscosities of solutions of K-PAM (potassium polyacrylamide), HEC (hydroxyethyl cellulose), PAC-LV (polyanionic cellulose), and FA-367 (polyacrylamide) at 1 s^−1^ were 3188.92 mPa.s, 47,030.48 mPa.s, 751.17 mPa.s, and 3284.53 mPa.s, respectively. After the photocatalytic degradation of the polymer by 0.5S-BiOBr_0.5_Cl_0.5_, it was almost the same as that of water. The PAC-LV, FA-367, and K-PAM degradation efficiencies of 0.5S-BiOBr_0.5_Cl_0.5_ exceeded 79% within 24 h (Figure 9e).

### 2.10. The Photocatalytic Mechanism of xS-BiOBr_0.5_Cl_0.5_ Materials

**Figure 10:**
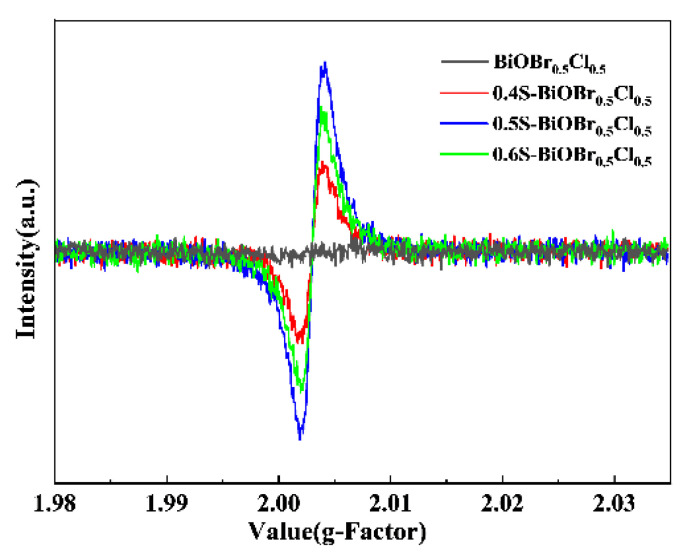
EPR spectra of BiOBr_0.5_Cl_0.5_ and xS-BiOBr_0.5_Cl_0.5_ materials.

The EPR spectra of the xS-BiOBr_0.5_Cl_0.5_ materials (Figure 10) indicated that oxygen vacancies played an important role in the visible-light-driven photocatalytic activity of the materials. The signal at a g-factor = 2.004 indicated electron trapping by the surface oxygen vacancies [51,52,53], and this signal was not detected in the EPR spectrum of BiOBr_0.5_Cl_0.5_. In comparison, distinct signals at a g-factor = 2.003 were detected in the EPR spectra of the xS-BiOBr_0.5_Cl_0.5_ materials, demonstrating the formation of oxygen vacancies on the surface of the materials [54]. Among the xS-BiOBr_0.5_Cl_0.5_ materials, 0.5S-BiOBr_0.5_Cl_0.5_ showed the highest intensity of the signal at a g-factor = 2.003, proving that its surface captured more electrons and contained more oxygen vacancies, which was in line with the XPS results.

Photocatalysts can generate electron–hole pairs under excitation by visible light. The holes (h^+^) can react with OH^−^ to produce ··OH, while the electrons (e^−^) can react with O_2_ to produce ^1^O_2_ and O_2_^−^. The h^+^,··OH, ^1^O_2_, and O_2_^−^ can participate as the active species in the photocatalytic degradation of pollutants.

**Figure 11:**
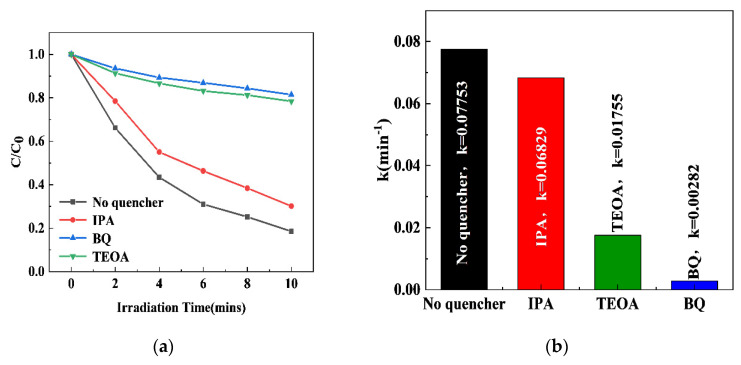
Efficiencies (**a**) and apparent rate constants (**b**) for the degradation of RhB by 0.5S-BiOBr_0.5_Cl_0.5_ in the absence and presence of active-species-capturing agents.

The participation of the above active species in the photocatalytic degradation of RhB by 0.5S-BiOBr_0.5_Cl_0.5_ was investigated through active-species-capturing experiments. BQ, IPA, and TEOA were used as the capturing agents for O_2_^−^, ·OH, and h^+^, respectively [55], and the effects of the capturing agents on the efficiency and apparent rate constant for the degradation of RhB by 0.5S-BiOBr_0.5_Cl_0.5_ were investigated. As shown in Figure 11a, compared with the RhB degradation efficiency of 0.5S-BiOBr_0.5_Cl_0.5_ in the absence of the capturing agents, the RhB degradation efficiencies of the material in the presence of BQ, IPA, and TEOA decrease by 62.96%, 11.65%, and 59.87%, respectively. The apparent rate constants for the degradation of RhB by 0.5S-BiOBr_0.5_Cl_0.5_ in the presence of BQ, IPA, and TEOA were 0.00282/min, 0.06829/min, and 0.01755/min, respectively (Figure 11b), suggesting that O_2_^·−^ and h^+^ were the main active species in the photocatalytic degradation of RhB by 0.5S-BiOBr_0.5_Cl_0.5_, with ·OH having a low contribution to the photocatalytic degradation of RhB.

**Figure 12:**
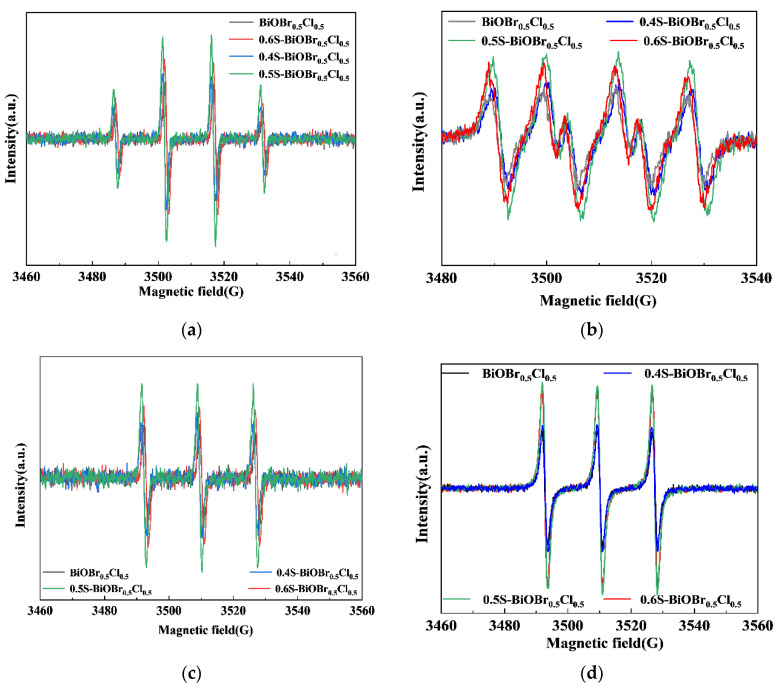
DMPO–·OH (**a**), DMPO–O_2_^·−^ (**b**), DMPO–h^+^ (**c**), and DMPO–^1^O_2_ (**d**) EPR spectra for BiOBr_0.5_Cl_0.5_ and xS-BiOBr_0.5_Cl_0.5_ materials.

The amounts of ·OH, O_2_^−^, h^+^, and ^1^O_2_ generated in the photocatalytic degradation of RhB by the xS-BiOBr_0.5_Cl_0.5_ materials were examined through an EPR analysis. As shown in Figure 12a,b, the intensity ratios of the signals in the DMPO (5,5-Dimethyl-1-pyrroline N-oxide)–·OH and DMPO–O_2_^−^ EPR spectra of the xS-BiOBr_0.5_Cl_0.5_ materials were 1:2:2:1 and 1:1:1:1, respectively, indicating that ·OH and O_2_^−^ were produced during the photocatalytic degradation of RhB by the materials. The order of the BiOBr_0.5_Cl_0.5_ and xS-BiOBr_0.5_Cl_0.5_ materials determined on the basis of the amounts of ·OH and O_2_^−^ produced by the photocatalysts was 0.5S-BiOBr_0.5_Cl_0.5_ > 0.6S-BiOBr_0.5_Cl_0.5_ > 0.4S-BiOBr_0.5_Cl_0.5_ > BiOBr_0.5_Cl_0.5_. Figure 12c,d show the amounts of h^+^ and ^1^O_2_ produced during the photocatalytic degradation of RhB by the BiOBr_0.5_Cl_0.5_ and xS-BiOBr_0.5_Cl_0.5_ materials. The order of the photocatalysts determined on the basis of the amounts of h^+^ and ^1^O_2_ they produced was 0.5S-BiOBr_0.5_Cl_0.5_ > 0.6S-BiOBr_0.5_Cl_0.5_ > 0.4S-BiOBr_0.5_Cl_0.5_ > BiOBr_0.5_Cl_0.5_. These results were in agreement with the results of the experiment on the photocatalytic degradation of RhB.

**Figure 13:**
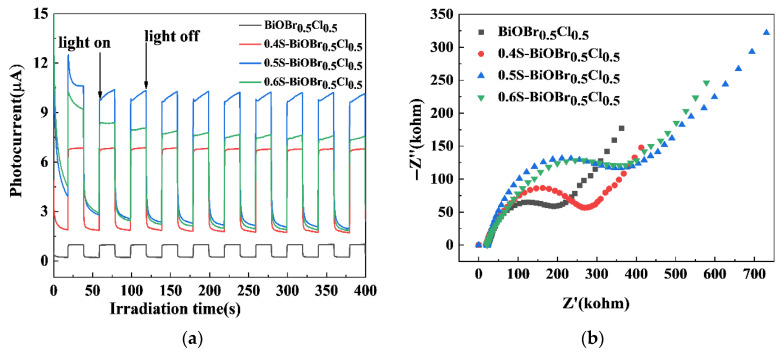
TPC spectra (**a**) and Nyquist plots (**b**) of BiOBr_0.5_Cl_0.5_ and xS-BiOBr_0.5_Cl_0.5_ materials.

Measurement of the TPC responses and electrochemical impedance is a direct method for verifying the efficiency of charge carrier separation in a photocatalyst. Under dark conditions, neither the BiOBr_0.5_Cl_0.5_ or xS-BiOBr_0.5_Cl_0.5_ material generated any TPC response (Figure 13a). On the other hand, under visible light irradiation, the photocurrent intensity of the xS-BiOBr_0.5_Cl_0.5_ materials gradually increased with an increasing concentration of sulfur dopants in the materials, with 0.5S-BiOBr_0.5_Cl_0.5_ exhibiting the highest photocurrent intensity among the materials, implying that the presence of sulfur dopants and oxygen vacancies in 0.5S-BiOBr_0.5_Cl_0.5_ enabled the material to prevent e^−^–h^+^ complexation and accelerate the charge transfer [56]. The Nyquist plots of the BiOBr_0.5_Cl_0.5_ and xS-BiOBr_0.5_Cl_0.5_ materials, which were obtained through an electrochemical impedance spectroscopy (EIS) analysis, are shown in Figure 13b. The smaller the radius of the arc in the Nyquist plot of a sample (BiOBr_0.5_Cl_0.5_, 0.4S-BiOBr_0.5_Cl_0.5_, 0.5S-BiOBr_0.5_Cl_0.5_, or 0.6S-BiOBr_0.5_Cl_0.5_), the better the sample hindered the complexation of photogenerated charge carriers, and the higher the photocatalytic efficiency of the sample. Compared with the Nyquist plot for BiOBr_0.5_Cl_0.5_, those for the xS-BiOBr_0.5_Cl_0.5_ materials showed smaller arc radii, with the Nyquist plot for 0.5S-BiOBr_0.5_Cl_0.5_ showing the smallest arc radius, suggesting that 0.5S-BiOBr_0.5_Cl_0.5_ had the lowest electron transfer resistance and the highest photocatalytic efficiency among the materials.

**Figure 14:**
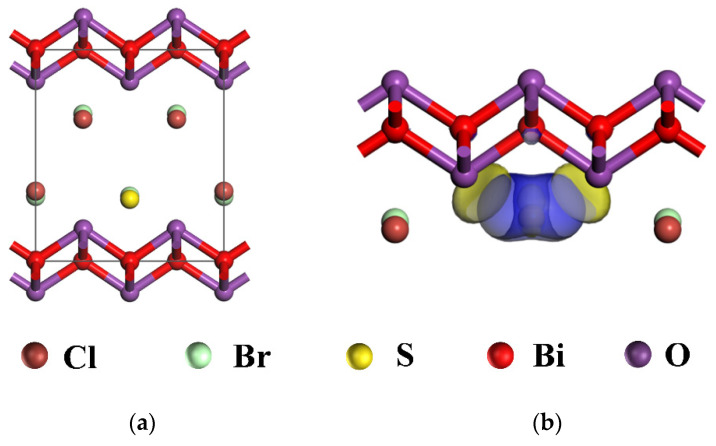
The cell model (**a**) and the electron-cloud profile (**b**) of 0.5S-BiOBr_0.5_Cl_0.5_.

DFT-based first-principles electronic structure calculations were performed on the electron-cloud structure of 0.5S-BiOBr_0.5_Cl_0.5_. The model for a cell of BiOBr_0.5_Cl_0.5_ was constructed using the following structural parameters: a = b = 3.891 Å, c = 8.092 Å, α = β = γ = 90°, and V_0_ = 11.08 Å. As shown in Figure 14a, the cell of BiOBr_0.5_Cl_0.5_ is a 2 × 1 × 1 cell containing 12 atoms, with sulfur atoms substituting the chloride atoms at the center of the cell. On the basis of the model constructed for the BiOBr_0.5_Cl_0.5_ cell, the distribution of the interlayer charges was analyzed through DFT-based first-principles calculations. A high charge density was formed between [Bi_2_O_2_]^2+^ and the atomic-sulfur layers (Figure 14b) due to the above substitution of chloride atoms with sulfur atoms (the electronegativity of the sulfur atoms was lower than that of the chloride atoms). The strong Bi–S bond accumulated electrons on the sulfur atoms and depleted electrons on the bismuth atoms, leading to polarization between the [Bi_2_O_2_]^2+^ and atomic-sulfur layers. This polarization enhanced the internal electric field and acted as a driving force for e^−^–h^+^ separation and transport, leading to improved photocatalytic efficiency in 0.5S-BiOBr_0.5_Cl_0.5_.

**Figure 15:**
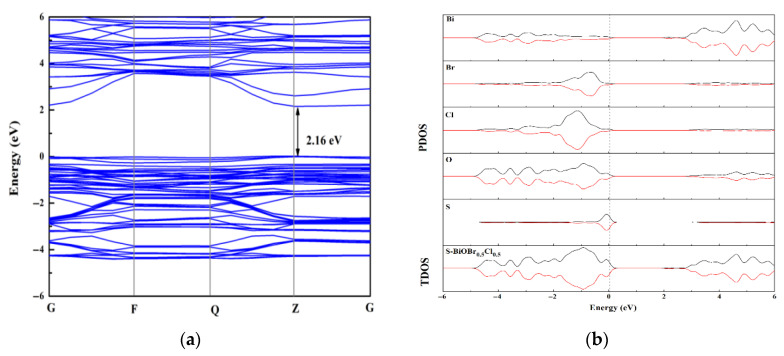
The bandgap profile (**a**) and state density (**b**) of 0.5S-BiOBr_0.5_Cl_0.5_.

The bandgap of 0.5S-BiOBr_0.5_Cl_0.5_ calculated through DFT-based first-principles calculation was 2.16 eV (Figure 15a), which was lower than that measured through UV–vis spectroscopy (2.47 eV). The difference between the calculated and measured bandgaps was due to the limitation of GGA-PBE, but this limitation did not affect the calculated results for the 0.5S-BiOBr_0.5_Cl_0.5_ electronic structure. The effective mass and mobility of charge carriers are directly related to the curvature of the energy-band structure, and the energy-band structure of 0.5S-BiOBr_0.5_Cl_0.5_ showed highly dispersed electronic energy levels at the bottom of the conduction band of the material, indicating that the material had a high charge carrier separation capacity and many photogenerated e^−^ that could participate in a photocatalytic reaction. Total density of states (TDOS) and partial density of states (PDOS) maps of 0.5S-BiOBr_0.5_Cl_0.5_ are shown in Figure 15b. The top of the valence band of 0.5S-BiOBr_0.5_Cl_0.5_ was dominated by the S 2p state, which caused it to span across the Fermi energy level without inducing any change to the bottom of the conduction band of the material. On the other hand, the bottom of the conduction band of 0.5S-BiOBr_0.5_Cl_0.5_ was dominated by the Bi 6p state. Therefore, it was inferred that e^−^ jumped from the S 2p hybridized orbitals at the top of the valence band of 0.5S-BiOBr_0.5_Cl_0.5_ to the unoccupied Bi 6p orbitals at the bottom of the conduction band of the material, improving the visible light response of the material.

**Figure 16:**
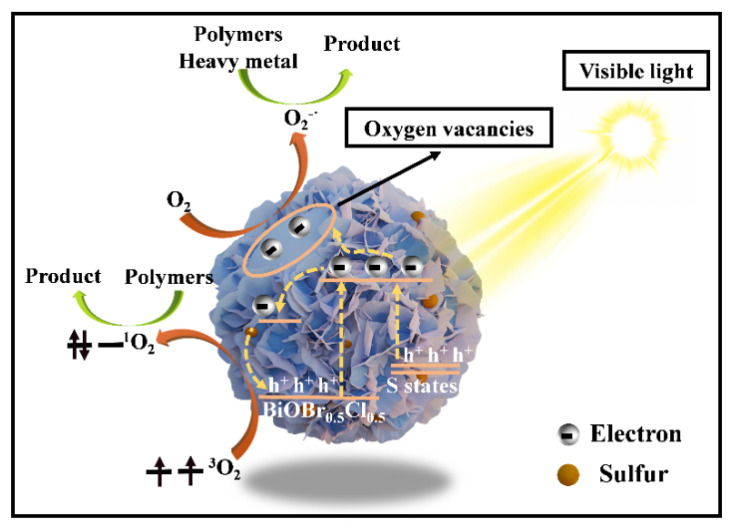
The photocatalytic mechanism of 0.5S-BiOBr_0.5_Cl_0.5_.

The photocatalytic mechanism of 0.5S-BiOBr_0.5_Cl_0.5_ is shown in Figure 16. BiOBr_0.5_Cl_0.5_ reacted with thiourea to generate abundant oxygen vacancies on the surface of 0.5S-BiOBr_0.5_Cl_0.5_. The oxygen vacancies not only captured photogenerated e^−^ but also facilitated the generation of active species (^1^O_2_ and O_2_^−^) on the surface of the material. Although the conduction-band position of 0.5S-BiOBr_0.5_Cl_0.5_ was similar to that of BiOBr_0.5_Cl_0.5_, the valence-band position of 0.5S-BiOBr_0.5_Cl_0.5_ was elevated relative to that of BiOBr_0.5_Cl_0.5_, which reduced the bandgap of 0.5S-BiOBr_0.5_Cl_0.5_ and improved the visible light response of the material. Moreover, the presence of sulfur dopants enhanced the internal electric field of 0.5S-BiOBr_0.5_Cl_0.5_, hindering the complexation of photogenerated e^−^ and h^+^. It was concluded that the high photocatalytic activity of 0.5S-BiOBr_0.5_Cl_0.5_ was contributed by the synergistic effect of multiple factors.

## 3. Conclusions

In this study, sulfur-doped and oxygen-vacancy-modified three-dimensional flower-like xS-BiOBr_0.5_Cl_0.5_ materials were prepared through hydrothermal synthesis. Sulfur doping increased the valence bands and decreased the bandgaps of the xS-BiOBr_0.5_Cl_0.5_ materials, improving the visible light response of the materials. Moreover, it induced polarization between [Bi_2_O_2_]^2+^ and the atomic-sulfur layers, enhancing the internal electric field and promoting e^−^–h^+^ separation. Furthermore, it generated abundant oxygen vacancies on the surface of the materials, which was favorable for capturing the photogenerated e^−^. Among the xS-BiOBr_0.5_Cl_0.5_ materials, 0.5S-BiOBr_0.5_Cl_0.5_ showed the best photocatalytic performance. It showed excellent photocatalytic degradation efficiencies for RhB (up to 91%), heavy-metal ions (up to 96%), and oilfield polymers (up to 79%). The xS-BiOBr_0.5_Cl_0.5_ materials can degrade organic molecules and heavy metals without causing secondary pollution, and they can be reused. Thus, they can be used for the low-cost and harmless treatment of waste drilling fluids, promoting the green development of the oil and gas industry. However, in practical implementation, the application of solar energy—as a renewable source—to treating waste drilling fluids poses significant challenges. Specifically, the inherent opacity and high viscosity of the gel matrix, combined with inadequate light penetration into the photocatalyst particles, result in a low electron transfer efficiency, leading to sluggish reaction kinetics and substantially prolonged treatment durations.

## 4. Materials and Methods

### 4.1. Materials

Bismuth(III) nitrate pentahydrate (Bi(NO_3_)_3_∙5H_2_O)(≥99%), potassium bromide (KBr) (≥99%), potassium chloride (KCl) (≥99%), thiourea, rhodamine B (RhB) (≥98%), benzoquinone (BQ) (≥99%), isopropanol (IPA) (≥99%), and triethanolamine (TEOA) (≥99%) were purchased from Anaiji Chemical Co., Ltd. (Shanghai, China). Deionized water was used in all experiments. The reagents were used without further purification.

### 4.2. Synthesis of the xS-BiOBr_0.5_Cl_0.5_ Materials

**Figure 17:**
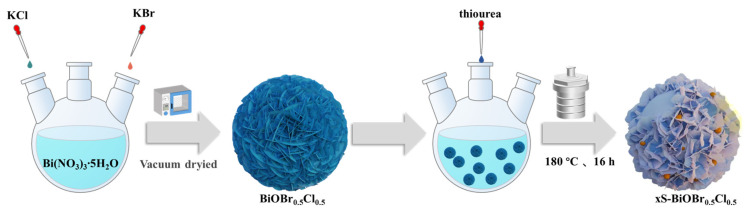
A schematic of xS-BiOBr_0.5_Cl_0.5_ synthesis via hydrothermal treatment.

The synthesis method of xS-BiOBr_0.5_Cl_0.5_ is shown in Figure 17. Bi(NO_3_)_3_·5H_2_O (3 mmol) and deionized water (50 mL) were added to a 100 mL three-necked round-bottom flask, and the mixture was stirred at 2000 rpm to form a Bi(NO_3_)_3_ solution. Nitrogen was injected to remove trapped air and maintain an anaerobic environment. Separately, 1.5 mmol of KBr and 1.5 mmol of KCl were dissolved in 20 mL of deionized water, and the resulting solution was slowly dripped into the Bi(NO_3_)_3_ solution using a 20 mL syringe. The mixture was stirred at 2000 rpm for 30 min and filtered, and the solid product was washed three times with deionized water and ethanol, followed by vacuum-drying at 60 °C for 12 h to obtain 3D flower-like BiOBr_0.5_Cl_0.5_. For the sulfur-doped materials, BiOBr_0.5_Cl_0.5_ (1 mmol) was dispersed in 20 mL of deionized water in a three-necked flask, stirred at 2000 rpm for 30 min, and purged with nitrogen. A specified amount of thiourea (0.4, 0.5, or 0.6 mmol) was added, and the mixture was transferred into a polytetrafluoroethylene-lined autoclave for hydrothermal treatment at 180 °C for 16 h. After cooling, the product was filtered, washed with deionized water and ethanol, and vacuum-dried at 60 °C for 12 h to yield xS-BiOBr_0.5_Cl_0.5_ (x = 0.4, 0.5, or 0.6).

### 4.3. Characterization of the xS-BiOBr_0.5_Cl_0.5_ Materials

The crystal structure and crystal phases of the xS-BiOBr_0.5_Cl_0.5_ materials were characterized using a D8 Advance X-ray diffractometer from BRUKER company (Billerica, MA, USA) (XRD) with a graphite-monochromatized Cu-K_α_ radiation source (λ = 0.15418 nm). The morphology of the materials was characterized using a Quanta 200F field-emission scanning electron microscope from FEI company (Hillsborough, OR, USA) (SEM) and a Tecnai G2 F30 high-resolution transmission electron microscope from FEI company (Hillsborough, OR, USA) (HRTEM). The thickness of the materials was measured using a Bioscope Resolve atomic force microscope from BRUKER company (Billerica, MA, USA) (AFM). The valence states of the surface chemical elements of the materials were analyzed using a K-Alpha X-ray photoelectron spectrometer from Thermo Fisher Scientific(Waltham, MA, USA). The light absorption characteristics and bandgaps of the materials were measured using a UV-2600 ultraviolet–visible (UV–vis) spectrometer from Shimadzu (Kyoto, Japan) in the wavelength range of 230–800 nm, with barium sulfate used as the standard. An EMXplus electron paramagnetic resonance (EPR) spectrometer (Bruker, USA) was used to characterize the roles of the superoxide anions (O_2_^−^), singlet oxygen (^1^O_2_), hydroxyl radicals (·OH), photogenerated holes, and oxygen vacancies in the photocatalytic degradation activity of the materials. The transient photocurrent (TPC) spectra and Nyquist plots were collected using a CHI660D electrochemical work station from CHI Instruments (Shanghai, China).

### 4.4. The Photocatalytic Degradation of RhB, Drilling Fluid Polymer Additives, and Heavy Metals

Photocatalytic degradation of RhB: An xS-BiOBr_0.5_Cl_0.5_ material (50 mg) was added to a 20 mg/L aqueous RhB solution (50 mL), and the suspension was magnetically stirred at 2000 rpm under dark conditions for 30 min to establish adsorption–desorption equilibrium between RhB and the photocatalyst. Then, the suspension was illuminated with a 0.21 W/cm^2^ intensity using a 300 W xenon lamp equipped with a UV-cut filter (λ > 400 nm; the xenon lamp was used as a visible light source). A sample was collected from the reactor every 2 min, and it was centrifuged at 8000× *g* rpm for 2 min. The supernatant was collected, and its UV–vis absorption spectrum was recorded in the wavelength range of 200–800 nm.

Photocatalytic degradation of polymers: xS-BiOBr_0.5_Cl_0.5_ material (1 g) was added to a polymer solution (50 mL), and the suspension was magnetically stirred for 30 min under dark conditions to establish adsorption–desorption equilibrium between the polymer and the photocatalyst. Then, the polymer solution was illuminated with a 300 W xenon lamp for 24 h. The chemical oxygen demand (COD) values of the polymer solution before and after photocatalytic degradation were measured.

Photocatalytic reduction of heavy metals: Potassium dichromate, chromium sulfate, lead nitrate, and mercury nitrate were used as the heavy-metal sources. The initial concentrations of the potassium dichromate, chromium sulfate, lead nitrate, and mercury nitrate solutions were 1.2 mg/mL, 2.4 mg/mL, 0.5 mg/mL, and 1.0 mg/mL, respectively. xS-BiOBr_0.5_Cl_0.5_ material (1 g) was added to the above potassium dichromate, chromium sulfate, lead nitrate, or mercury nitrate solution (50 mL), and the suspension was magnetically stirred for 30 min under dark conditions to establish adsorption–desorption equilibrium between the heavy-metal ions and the photocatalyst. Photocatalytic degradation of the heavy-metal ions was carried out for 96 h under illumination with a 300 W xenon lamp, and the concentration of the potassium dichromate, chromium sulfate, lead nitrate, or mercury nitrate solution after the photocatalytic degradation was tested using an atomic fluorescence spectrometer (Thermo Fisher Scientific, USA). The settings of the spectrometer were as follows: the atomization temperature was 200 °C, the liquid height was 8 mm, the carrier gas flow rate was 300 mL/min, the shielding gas flow rate was 800 mL/min, and the reading time was 10 s.

### 4.5. The Density Functional Theory (DFT) Computations

Cambridge Sequential Total Energy Package (CASTEP) was utilized to perform the DFT computations using the pseudopotential plane wave (PPW) method. Electron–ion interactions were described using ultrasoft pseudopotentials (USPs). A plane-wave basis set was employed to expand the wave functions with a kinetic energy cutoff of 500 eV. The functional parameterized by the Perdew–Burke–Ernzerhof general gradient approximation (GGA-PBE) was used to analyze the electron-exchange and electron-correlation interactions.

During geometry optimization, all atoms were allowed to relax. Brillouin-zone integration was conducted using a Monkhorst–Pack (MP) grid spacing of 0.08/Å. The convergence criterion for the electronic self-consistent field (SCF) loop was set to 10^−6^ eV/atom. The atomic structures were optimized until the residual forces were below 0.03 eV/Å.

## Data Availability

No new data were created or analyzed in this study.
